# Mel1c Mediated Monochromatic Light-Stimulated IGF-I Synthesis through the Intracellular G_α_q/PKC/ERK Signaling Pathway

**DOI:** 10.3390/ijms20071682

**Published:** 2019-04-04

**Authors:** Shujie Ning, Zixu Wang, Jing Cao, Yulan Dong, Yaoxing Chen

**Affiliations:** Beijing Advanced Innovation Center for Food Nutrition and Human Health, College of Veterinary Medicine, China Agricultural University, Haidian, Beijing 100193, China; ningshujie@126.com (S.N.); zxwang2007@163.com (Z.W.); caojing315@126.com (J.C.); ylbcdong@163.com (Y.D.)

**Keywords:** IGF-I, monochromatic light, melatonin, extracellular regulated protein kinases, G_α_q, protein kinase C

## Abstract

Previous studies have demonstrated that monochromatic light affects plasma melatonin (MEL) levels, which in turn regulates hepatic insulin-like growth factor I (IGF-I) secretion via the Mel1c receptor. However, the intracellular signaling pathway initiated by Mel1c remains unclear. In this study, newly hatched broilers, including intact, sham operation, and pinealectomy groups, were exposed to either white (WL), red (RL), green (GL), or blue (BL) light for 14 days. Experiments in vivo showed that GL significantly promoted plasma MEL formation, which was accompanied by an increase in the MEL receptor, Mel1c, as well as phosphorylated extracellular regulated protein kinases (p-ERK1/2), and IGF-I expression in the liver, compared to the other light-treated groups. In contrast, this GL stimulation was attenuated by pinealectomy. Exogenous MEL elevated the hepatocellular IGF-I level, which is consistent with increases in cyclic adenosine monophosphate (cAMP), G_α_q, phosphorylated protein kinase C (p-PKC), and p-ERK1/2 expression. However, the Mel1c selective antagonist prazosin suppressed the MEL-induced expression of IGF-I, G_α_q, p-PKC, and p-ERK1/2, while the cAMP concentration was barely affected. In addition, pretreatment with Ym254890 (a G_α_q inhibitor), Go9863 (a PKC inhibitor), and PD98059 (an ERK1/2 inhibitor) markedly attenuated MEL-stimulated IGF-I expression and p-ERK1/2 activity. These results indicate that Mel1c mediates monochromatic GL-stimulated IGF-I synthesis through intracellular G_α_q/PKC/ERK signaling.

## 1. Introduction

The characteristics of light, including its source, spectra, intensity, and regimen, play important roles in avian growth and reproduction. Many studies have focused on the role of light spectra in chicken growth in recent years, finding that different wavelengths of light affected the growth of turkeys [1] and broilers [2,3]. Furthermore, green light (GL) stimulation has been shown to accelerate muscle growth in broilers [4], and enhance the secretion of insulin-like growth factor I (IGF-I) by chick embryo hepatocytes [5,6]. IGF-I has been shown to be a key regulator of muscle development and metabolism in birds and other vertebrate species [7,8], and promoted satellite cells proliferation through IGF-I signaling [9]. Liu et al. [10] have shown that GL illumination promoted chick satellite cell myogenic processes during early posthatch stages, and IGF-I played a central role. Melatonin (MEL), which is mainly synthesized by the pineal gland and associated with illumination, is a key indicator in photoelectric conversion [11,12,13,14]. Previous studies have shown that monochromatic light influenced the secretion of MEL [15], which was involved in IGF-I synthesis [5,16]. However, the molecular mechanism of the monochromatic light influence of IGF-I secretion in broilers is not fully understood.

Avian MEL receptors, including Mel1a, Mel1b, and Mel1c, belong to the heterotrimeric guanine nucleotide binding proteins (G protein)-coupled receptor (GPCR) family [17]. The G proteins G_α_s, G_α_i, and G_α_q have been reported to bind to melatonin receptors [18,19]. The binding of receptors to G_α_s leads to increased levels of cyclic adenosine monophosphate (cAMP), while the binding to G_α_i leads to a contrasting consequence. Additionally, G_α_q coupled receptors are associated with a rise in intracellular calcium as a result of phospholipase C (PLC) pathway activation [20]. Furthermore, G proteins stimulate distinct downstream effectors, including enzymes, ion channels, and small GTPases, thus regulating multiple signaling pathways, including those involved in the activation of mitogen-activated protein kinase (MAPK) modules [21,22,23,24]. In this study, we investigated the pathway of activation of monochromatic photostimulated IGF-I synthesis in broiler livers by Mel1c, and found that a signaling cascade of G_α_q/protein kinase C (PKC)/extracellular regulated protein kinases (ERK) is involved.

## 2. Results

### 2.1. Monochromatic Green Light-Regulated Hepatic IGF-I Expression through Mel1c

**Figure 1:**
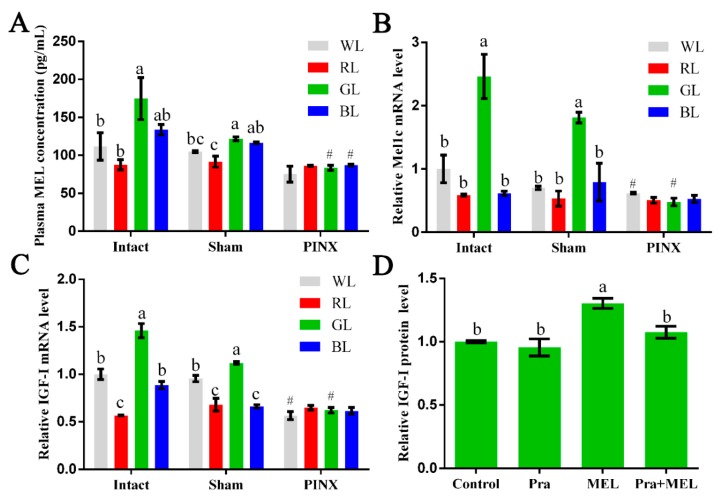
Effect of Mel1c on insulin-like growth factor I (IGF-I) synthesis in the livers of broilers following monochromatic light stimulation. Plasma and livers from broilers (*n* = 4) of each operative group were collected at P14 following exposure to different light treatments. (**A**) The concentration of melatonin (MEL) in plasma was detected by ELISA. Relative mRNA levels of Mel1c (**B**) and IGF-I (**C**) in the liver were detected by QRT-PCR. (**D**) Hepatocytes were isolated from GL-treated intact broilers (*n* = 5) at P14 and were incubated with 250 pg/mL of MEL, 1 µM of prazosin, or a combination of the two for 24 h. Then, the cell supernatant was collected for IGF-I protein expression analysis by ELISA. Values within the same treatment group (Intact, Sham or PINX) with no common letters (a, b or c) are significantly different with each other (*p* < 0.05). Relative Mel1c and IGF-I mRNA or protein levels were quantified using the intact WL group or control as 100%. # *p* < 0.05 compared with corresponding light treatments in the sham group. Pra, prazosin; WL, white light; RL, red light; GL, green light; BL, blue light; Sham, sham operation; PINX, pinealectomy operation.

In this study, following exposure to different light treatments, the plasma and livers of intact, sham, or pinealectomy broilers were collected for measurement of the MEL concentration as well as Mel1c and IGF-I mRNA detection at P14. The MEL concentration in plasma in the intact GL group was 56.47% and 99.35% significantly higher than the white (WL) (*p* = 0.020) and red (RL) (*p* = 0.003) groups, and slightly higher than the blue (BL) (30.68%, *p* = 0.106) group, respectively (Figure 1A). Accordingly, the mRNA levels of Mel1c (Figure 1B) and IGF-I (Figure 1C) in the livers of intact chicks exposed to GL were increased by 146.12–320.39% (*p* = 3.41 × 10^−5^–3.17 × 10^−4^) and 45.91–157.89% (*p* = 3.46 × 10^−8^–3.64 × 10^−5^), respectively. After pinealectomy, the MEL concentration in the plasma of the GL and BL groups were significantly decreased by 31.39% (*p* = 1.66 × 10^−4^) and 25.08% (*p* = 2.86 × 10^−6^), and slightly decreased in WL and RL, by 28.27% (*p* = 0.084) and 5.99% (*p* = 0.545), respectively, compared with the concentrations of the sham operation group members following corresponding light treatments (Figure 1A). Consistent with the decrease in the MEL level in plasma, the mRNA levels of Mel1c (Figure 1B) and IGF-I (Figure 1C) in the livers of the pinealectomy groups under GL treatment were reduced by 73.61% (*p* = 1.45 × 10^−5^) and 44.51% (*p* = 4.93 × 10^−5^), respectively. Furthermore, there were no statistically significant differences in the MEL, Mel1c, or IGF-I levels among various monochromatic light treatment groups after the pinealectomy operation. These results indicate that monochromatic green light might regulate IGF-I expression through MEL and Mel1c.

To further determine the relationship between MEL and Mel1c receptors with monochromatic light-induced hepatic IGF-I expression, liver cells from GL-treated broilers were isolated at P14. As Figure 1D shows, treatment of isolated primary hepatocytes with 250 pg/mL of melatonin for 24 h resulted in a 1.3-fold (*p* = 0.0003) increase in IGF-I protein expression compared with the control. When the effect of melatonin was abrogated by the pretreatment of the Mel1c antagonist (prazosin), this resulted in no obvious differences in the control and prazosin-alone groups (Figure 1D). These results indicate that monochromatic GL regulates IGF-I protein expression through Mel1c.

### 2.2. G_α_q Coupled to Mel1c is Involved in MEL-Induced IGF-I Expression

**Figure 2:**
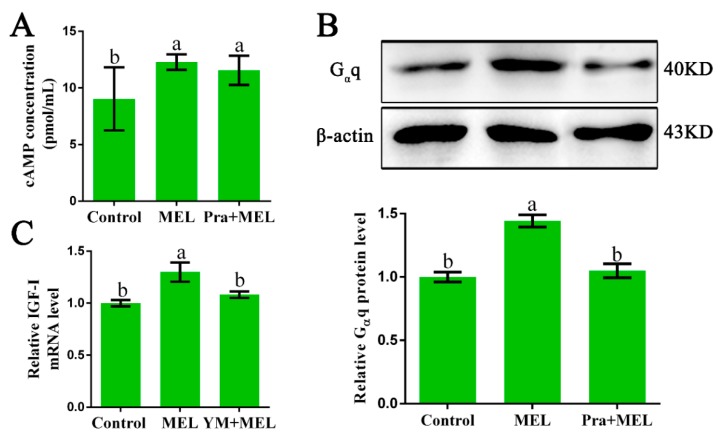
Identification of the G protein coupled to Mel1c that is involved in IGF-I expression. Hepatocytes were isolated from GL-treated intact broilers at P14 and incubated with 250 pg/mL of MEL, 1 µM of prazosin, or a combination of the two for 24 h. Then, the cells were collected for cAMP analysis by ELISA (**A**), and G_α_q analysis by Western blot (**B**). (**C**) MEL-treated cells were co-incubated with or without 10 µM of Ym254890 for 24 h, and then collected to detect IGF-I mRNA expression by QRT-PCR. The relative G_α_q protein level was quantified by the density ratio of G_α_q protein to β-actin protein. Relative values were quantified using the control as 100%. Values with no common letters (a, b) are significantly different with each other (*p* < 0.05). Pra, prazosin; YM, Ym254890 (blocks the exchange of GDP for GTP in G_α_q/11 activation); MEL, melatonin.

In order to explore which G proteins could couple to Mel1c and mediate the effect of MEL on IGF-I synthesis, hepatocytes were isolated from GL-treated intact broilers at P14, and the potential involvement of G_α_s, G_α_i, and G_α_q was investigated. The effects of melatonin in the presence or absence of a Mel1c antagonist (prazosin) on intracellular cAMP changes in primary hepatocytes were assessed by ELISA. The results showed that 250 pg/mL of melatonin induced a 1.36-fold increase (*p* = 0.015) in the intracellular cAMP level in primary hepatocytes as compared with the control (Figure 2A). The elevation of intracellular cAMP levels induced by melatonin was scarcely affected by 1 µM of prazosin, which is a Mel1c antagonist (Figure 2A). The data suggest that Mel1c might not couple with G_α_i or G_α_s in liver cells.

Then, the protein level of G_α_q in response to additional melatonin in the presence or absence of a Mel1c antagonist (prazosin) in primary hepatocytes from GL-treated intact broilers was measured by Western blot analysis. G_α_q protein expression was detected in the cell lysates using a primary antibody against G_α_q. Treatment of isolated primary hepatocyte cells with 250 pg/mL of melatonin for 24 h resulted in a 1.44-fold (*p* = 2.77 × 10^−5^) increase in G_α_q protein expression (Figure 2B) and a 1.30-fold (*p* = 0.008) increase in IGF-I mRNA expression (Figure 2C). The observed melatonin-induced upregulation in G_α_q protein expression was abrogated by 1 µM of Mel1c antagonist, and the MEL-induced increase of IGF-I mRNA expression was abolished by Ym254890, which is a G_α_q protein inhibitor. These results indicate that G_α_q might couple to Mel1c and be involved in MEL-induced IGF-I expression in the livers of GL-treated broilers.

### 2.3. Mel1c-Activated PKC through G_α_q Involved in MEL-Induced IGF-I Expression

**Figure 3:**
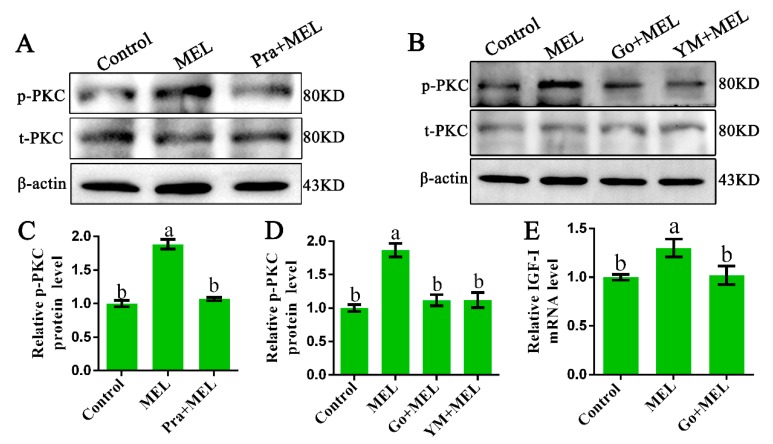
Role of protein kinase C (PKC) in Mel1c regulation of IGF-I expression in isolated hepatocytes. Hepatocytes were isolated from GL-treated intact broilers at P14 and incubated with 250 pg/mL of MEL in the presence or absence of 1 µM of prazosin for 24 h to detect the phosphorylated PKC level by Western blot (**A**,**C**). Primary hepatocytes treated with 250 pg/mL of MEL were incubated with or without 10 µM of Go9863 or Ym254890 for 24 h to detect the phosphorylated PKC level by Western blot (**B**,**D**), and IGF-I mRNA expression was assessed by QRT-PCR assay (**E**). The relative phosphorylated protein level was quantified by its ratio to the total protein density level. The relative values were quantified using the control as 100%. Values with no common letters (a, b) are significantly different with each other (*p* < 0.05). Pra, prazosin; p-PKC, phosphorylated PKC; t-PKC, total PKC; Go, Go9863 (a pan PKC inhibitor); YM, Ym254890 (blocks the exchange of GDP for GTP in Gq/11 activation); MEL, melatonin.

The α-subunits of G_α_q transduce signals from their cognate receptors to specific cellular responses via the activation of the effector PLCβ and protein kinase C (PKC) [25]. Thus, we investigated whether PKC is involved in Mel1c-induced IGF-I activation. The protein levels of the phosphorylated and total PKC in response to the presence of additional melatonin in primary hepatocytes in the presence or absence of a Mel1c antagonist (prazosin) were measured by Western blot analysis. The treatment of isolated primary hepatocyte cells with melatonin for 24 h resulted in a 1.88-fold (*p* = 5.05 × 10^−8^) increase in relative activated PKC protein as compared with the control (Figure 3A,C). The observed melatonin-induced upregulation in activated PKC protein expression was abrogated by 1 µM of Mel1c antagonist (prazosin). Additionally, MEL-induced PKC expression phosphorylation was also abrogated by 10 µM of Ym254890 (G_**α**_q antagonist) or 10 µM of Go9863 (PKC inhibitor) (Figure 3B,D). The data suggest that G_**α**_q couples to excite Mel1c and activate PKC signaling in the isolated hepatocytes of GL-treated broilers. Furthermore, MEL-induced upregulation of IGF-I mRNA expression was blocked when the cells were co-incubated with Go9863 (a PKC inhibitor) in isolated hepatocyte cells (Figure 3E). Together, the results indicate that G_**α**_q/PKC is involved in the process of Mel1c, mediating the effects of melatonin on IGF-I mRNA expression in hepatic cells.

### 2.4. ERK1/2 Participates in G_α_q-Coupled Mel1c-Mediated IGF-I Synthesis

**Figure 4:**
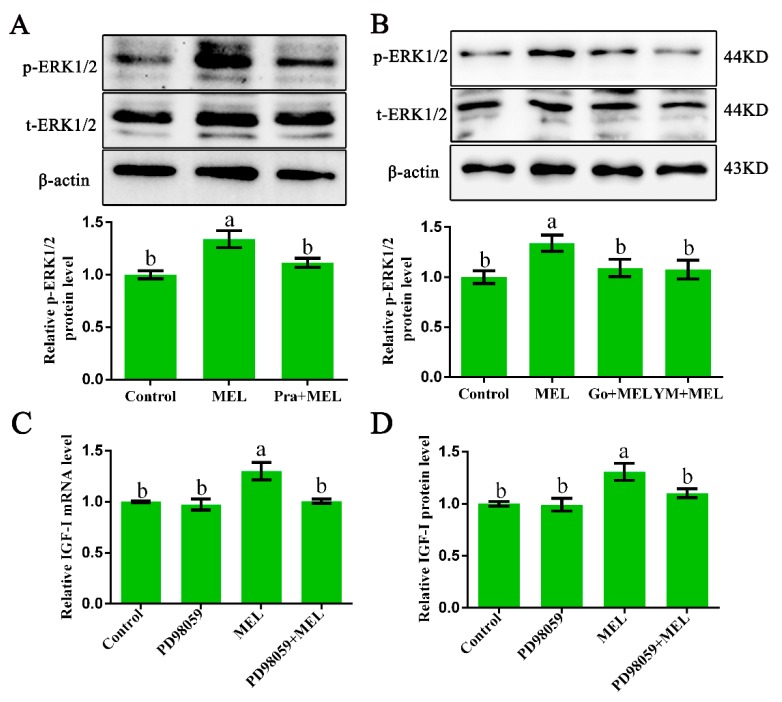
Relevance of ERK1/2 activation to IGF-I expression in isolated hepatocytes. (**A**,**B**) Phosphorylated ERK1/2 protein expression in hepatocytes. Hepatocytes were isolated from GL-treated intact broilers at P14. The cells were incubated with 250 pg/mL of MEL in the presence or absence of 1 µM of prazosin, 10 µM of Go9863, or 10 µM of Ym254890 for 24 h, and then phosphorylated ERK1/2 protein expression was assessed by Western blot. The relative phosphorylated ERK1/2 protein level was quantified by the ratio of phosphorylated to total ERK1/2 protein density using the control as 100%. (**C**,**D**) Relative IGF-I mRNA and protein expression in hepatocytes. Cultured hepatocytes were incubated with 250 pg/mL of MEL in the presence or absence of 10 µM of PD98059 for 24 h, and IGF-I mRNA and protein expression were assessed by QRT-PCR and ELISA assay, respectively. Relative IGF-I mRNA and protein levels were quantified using the control as 100%. Values with no common letters (a, b) are significantly different with each other (*p* < 0.05). p-ERK1/2, phosphorylated ERK1/2; t-ERK1/2, total ERK1/2; MEL, melatonin; Go, Go9863 (a pan PKC inhibitor); YM, Ym254890 (blocks the exchange of GDP for GTP in G_**α**_q/11 activation).

It is well established that G_α_q-activated PKC can stimulate ERK1/2 signaling [25,26]. Thus, ERK1/2 protein expression in primary hepatocytes from GL-treated broilers, cultured with or without MEL, in the presence or absence of 1 µM of prazosin, 10 µM of Ym254890 (G_**α**_q antagonist), or 10 µM of Go9863 (PKC inhibitor) for 24 h, was analyzed by Western blot analysis. The treatment of isolated primary hepatocyte cells with melatonin for 24 h resulted in a 1.34-fold (*p* = 0.001) increase in the phosphorylated ERK1/2 level as compared with that of the control. The observed melatonin-induced upregulation in p-ERK1/2 protein expression was abrogated by a Mel1c antagonist (Figure 4A) and G_**α**_q or PKC antagonists (Figure 4B). These results imply that Mel1c activates the ERK1/2 signaling pathway via a G_**α**_q/PKC cascade.

In addition, IGF-I mRNA and protein expression detection showed that the melatonin-induced increases in IGF-I mRNA and protein expression were abrogated by ERK1/2 inhibitor (Figure 4C,D), suggesting that ERK1/2 might participate in G_**α**_q-coupled Mel1c-mediated IGF-I synthesis.

**Figure 5:**
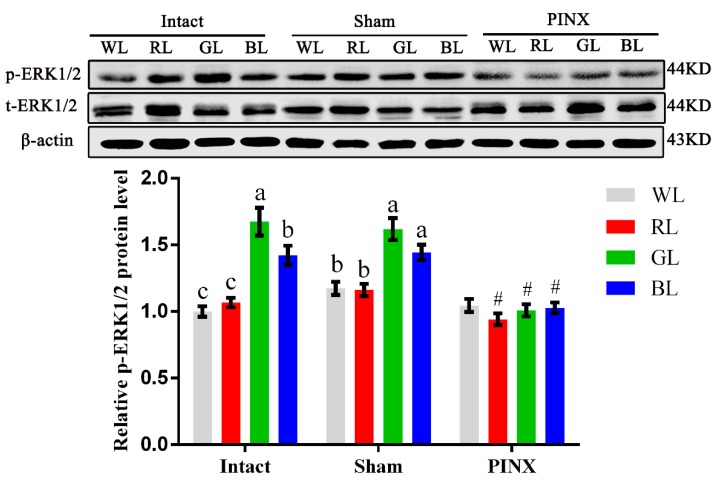
ERK1/2 activation levels in different light and operation treated broiler livers. Livers from broilers (*n* = 4) of each group were collected at P14, and phosphorylated and total ERK1/2 protein levels were assessed by Western blot in vivo. Values under different monochromatic light treatments within the same operational group (Intact, Sham or PINX) with no common letters (a, b or c) are significantly different with each other (*p* < 0.05). # *p* < 0.05 compared with corresponding light treatments in the sham group. The relative phosphorylated ERK1/2 protein level was quantified by the ratio of phosphorylated to total ERK1/2 protein density using the intact WL group as 100%. WL, white light; RL, red light; GL, green light; BL, blue light; PINX, pinealectomy; Sham, sham operated; p-ERK1/2, phosphorylated ERK1/2; t-ERK1/2, total ERK1/2.

To obtain further support for the involvement of the Mel1c/G_**α**_q/PKC/ERK1/2 cascade in the monochromatic light regulation of IGF-I synthesis in broiler livers, hepatic ERK1/2 activation of the intact, sham, and pinealectomy birds were studied following different light treatments by Western blot analysis in vivo. Exposure to monochromatic light affected hepatic ERK1/2 activation. The phosphorylated ERK1/2 level in intact GL broiler livers was 67.45% (*p* = 1.61 × 10^−5^), 57.01% (*p* = 4.34 × 10^−5^), and 17.74% (*p* = 0.024) higher than that of WL, RL, and BL in intact birds, respectively. Pinealectomy reduced phosphorylated ERK1/2 in the liver of RL, GL and BL groups by 10.97–37.67% (*p* = 0.001–0.014), respectively, compared with the corresponding light treatments in the sham operation group, and the differences among various monochromatic light treatment groups were not statistically significant (Figure 5). The trends of ERK1/2 activation levels in broilers following different light and operation treatments were consistent with the trends of Mel1c and IGF-I mRNA expression in the liver following different monochromatic light and operation treatments in vivo. The results indicate that the ERK1/2 cascade participates in monochromatic light induced-Mel1c mediated IGF-I expression.

## 3. Discussion

Previous studies have demonstrated that the monochromatic green light promotes the pineal gland releasing MEL [27] and improving hepatic IGF-I synthesis and secretion, which finally accelerated the broiler growth rate [3,4,6,28]. The increased IGF-I promoted satellite-cell proliferation and differentiation [6,9], which was responsible for muscle growth [29]. In this study, GL elevated plasma MEL secretion, and this was also accompanied by increases of Mel1c and IGF-I mRNA levels in the liver. The lower level of plasma MEL that resulted from pinealectomy led to Mel1c and IGF-I mRNA expression being decreased, indicating that MEL mediates monochromatic light-induced IGF-I synthesis. On the other hand, in vitro assays showed a dramatic reduction in MEL-induced hepatocyte IGF-I protein secretion when Mel1c was blocked. This study shows that MEL membrane receptor 1c is involved in monochromatic light-induced IGF-I secretion. The regulation of MEL in IGF-I synthesis has also been reported in human granulosa cells [30]. However, the intracellular signaling mechanism for Mel1c that transduces monochromatic light information into IGF-I synthesis still needs to be further deepened and explored.

In the present study, we found that the protein expression of Mel1c was upregulated by 250–2000 pg/mL melatonin treatment for 24 h (Figure S1). Melatonin could regulate its receptors’ expression for signal transduction [31,32,33,34]. The high level of Mel1c at 24 h might result in downstream signaling activation until 24 h. This research has referred to the predecessor in the experiment design regarding timing, such as the p-ERK activation being observed until 24 h after stimulation in breast cancer [35], NE-like LNCaP cells [36], and GC-2 cells [37], and the phosphorylated protein kinase C (p-PKC) activation at 24 h was also observed in ovarian surface epithelial cancer cells [38], GC-2 cells [37], podocytes [39], glial cells [40], and HT22 cells [41]. Thus, we perform signaling evaluation at 24 h post-treatment in vitro. MEL membrane receptors belonging to (G protein)-coupled receptors can couple with G_α_s, G_α_q, or G_α_i to transduce various signals related to MEL action [19,42,43]. To gain a better understanding of the signal transduction of melatonin in IGF-I synthesis in broiler livers, identification of the specific G proteins coupled to Mel1c for the relay of melatonin to downstream activation is important. It is well known that the coupling of receptors with G_α_s leads to the increased production of cyclic adenosine monophosphate (cAMP) via the adenylate cyclase pathway, but this process is inhibited by coupling with G_α_i. Additionally, the coupling of receptors with G_α_q primarily activates the membrane-associated phospholipase C and protein kinase C (PLC/PKC) pathway [21]. In this study, MEL induced an increase in the intracellular cAMP level, which was scarcely influenced by the Mel1c inhibitor. The enhancement of cAMP in response to MEL treatment might occur due to G_α_s coupling to Mel1a. For this reason, Mel1a has been reported to couple with G_α_s in COS-7 cells [44] and prostate cancer cells [19,45], and induce cAMP elevation via MEL. Although Mel1c has been reported to couple with G_α_i to inhibit cAMP formation in HEK293 cells [43], it seems that it does not couple with G_α_s or G_α_i in GL-treated chick liver cells. The apparent disparity in the mechanisms involved in Mel1c coupling to G proteins in chick liver cells can be partly explained by the different animal species and culture conditions used in various studies.

The detection of G_α_q mRNA or protein expression has been reported to evaluate the involvement of G_α_q in signal transduction. For instance, G_α_q mRNA expression was measured by quantitative real-time PCR, and the increased G_α_q mRNA expression or the activated G_α_q signaling was associated with cardiac hypertrophy, and G_α_q expression was inducible upon stimulation [46]. G_α_q protein expression was also analyzed with Western blot in K562 cells, and showed a slight increase within five days of treatment with hemin, compared with the untreated cells [47]. Levels of G proteins that were altered by stimulation [48] indicated that increased levels of G proteins might reflect signal transduction to some extent. In this study, the protein expression of G_α_q in cultured hepatocytes was enhanced by MEL, and the enhancement was abolished by a Mel1c inhibitor. It is classic that an agonist binding at the extracellular part of a GPCR induces conformational changes in cytoplasmic parts, forming an interactive interface for recruiting G proteins. However, a lot of studies suggest GPCRs and G-proteins forming a preassembled (or precoupled) complex [49,50,51,52]. Thus, we hypothesize that the high level of GPCR might result in an increase of the coupled G protein. That is to say, the elevated G_α_q protein might result from the increased Mel1c expression. This result suggests that Mel1c might couple with G_α_q and mediate the action of MEL. Further, it was shown that Mel1c can couple with G_α_q in transfected COS-7 cells [53]. MEL-induced IGF-I mRNA expression was also blocked by a G_α_q inhibitor, indicating that the activation of G_α_q proteins is involved in the signal transduction of Mel1c-mediated IGF-I synthesis in cultured chick liver cells. Accordingly, insulin secretion in human islets and transfected HEK293 cells could be mediated through G_α_q-coupled signaling [54,55].

G_α_q transduces signals from their cognate receptors to specific cellular responses via the activation of the effector PLCβ, which can activate protein kinase C (PKC) [25,26]. MEL membrane receptors have also been shown to stimulate the PLC/PKC pathway in the MEL signal transduction process [56,57,58]. In this study, MEL was found to phosphorylate PKC in hepatocytes. Moreover, blocking either Mel1c or G_α_q using specific inhibitors was enough to abolish the effects of MEL on PKC activation and IGF-I mRNA expression. There is evidence of the participation of PKC signaling in insulin secretion, that GLP-1-activated PKCs may contribute to insulin secretion INS-1 cells [59] and pancreatic islets [60,61]. These data indicate that activated PKC stimulated by G_α_q is involved in MEL-induced IGF-I expression, which is mediated by Mel1c.

Evidence has shown that a variety of different GPCRs exert their effects on cell growth and differentiation through the ERK1/2 cascade. Even though signaling pathways stimulated by different G proteins were intricate, most of them would activate ERK1/2 [62]. Thus, ERK1/2 is commonly used to measure the functional outcome of receptor stimulation [63]. In this regard, we explored ERK1/2 activation under the stimulation of Mel1c in cultured hepatocytes. There was a depression of MEL-induced phosphorylated ERK1/2 when Mel1c was blocked. In addition, MEL-induced phosphorylated ERK1/2 was also dismissed by G_α_q and PKC antagonists. Reports have shown that G_α_q-activated PKC could stimulate the ERK1/2 module by direct phosphorylation or by indirect C-Raf stimulation [25]. These results support the notion that MEL initiates the PKC/ERK signaling pathway through Mel1c coupling with G_α_q. Furthermore, the stimulation of both hepatocellular IGF-I mRNA and protein expression by MEL was obviously reduced by ERK1/2 inhibitor in vitro, indicating that ERK1/2 signaling also participates in MEL-induced IGF-I expression.

To further test the relevance of the G_α_q/PKC/ERK signal cascade in the process of hepatic IGF-I expression stimulated by monochromatic light, chick liver ERK1/2 activation was detected in vivo. Hepatic ERK1/2 activation was enhanced by GL stimulation and reduced by pinealectomy. The trends shown for ERK1/2 activation under monochromatic light stimulation were consistent with the expression of IGF-I mRNA, implying an involvement of ERK1/2 in monochromatic light that affects the IGF-I secretion process. These results indicate that ERK1/2 is involved in the monochromatic light-stimulated IGF-I expression process, which is consistent with a previous study [64] that found that the ERK, PI3K/Akt, and JAK2/STAT5 signaling pathways mediate the GH stimulation of IGF-I mRNA and protein expression. Although MEL was reported to be cytostatic for cancer cells through ERK1/2 signaling [36,65,66], the stimulation of MEL on the MEK/ERK1/2 pathway was also shown to mediate protective signaling transduction [67] and cell proliferation [68]. It seems that MEL is a pleiotropic molecule that protects the body from detrimental effects and is involved in the regulation of development. Collectively, these results indicate that Mel1c mediates GL-stimulated IGF-I synthesis through the intracellular G_α_q/PKC/ERK signaling pathway.

## 4. Materials and Methods

### 4.1. Ethics Statement

All of the animal experiments were in accordance with China Agricultural University (CAU) Institutional Animal Care and Use Committee guidelines (ID: CAU20171114-2). The animal experimental protocol was approved by the Animal Welfare Committee of CAU.

### 4.2. Animals and Treatment

Posthatching day (P) 0 Arbor Acre male broilers were procured from Beijing Huadu Breeding, P. R. China. The animals were raised in four separate colored light rooms. A light-emitting diode (LED, Hongli Tronic Co., Guangzhou, China) system was used as the unique light source in each room to generate white (WL, 400–700 nm, control group), red (RL, 660 nm), green (GL, 560 nm), or blue (BL, 480 nm) light [15,69]. All of the light sources were standardized by having an illuminance of 15 ± 0.2 lux (ST-85 model automatic range luminometer; Photoelectric Instrument Factory, Beijing, China) at the head of the broilers. In each light stimulation condition, the broilers were divided into the pinealectomy (*n* = 4), sham operation (*n* = 4), and intact groups (*n* = 4), with a further five intact birds in the GL group being used for in vitro assays. In each light-treatment group, pinealectomy and sham operations were performed at P3. Before each surgery, the birds were anesthetized with an intraperitoneal injection of Nembutal (30–40 μg per gram of body weight, 57-33-0, St. Louis, MO, USA) in accordance with the description by Karaganis [70], as follows. Anesthetized birds were secured with an avian stereotaxic apparatus. The skin, meninges, and skull were split to expose the pineal gland. The pineal gland was removed, and the opening was packed with gel foam to reduce bleeding. Then, the wound was closed with surgical sutures and treated with a topical antibiotic ointment. The sham surgeries were performed in exactly the same way, except that the pineal gland was left intact. After the operations, the birds were immediately returned to isolated cells with the correct corresponding light colors. The broilers were kept at a temperature of 32 °C during the first week, which was then reduced by 2 °C per day until it reached 26 °C. The relative humidity was maintained at 55–60%, with an alternative light/dark cycle (23 h light, 1 h dark; i.e., light from 1:00–23:00) to ensure that the animals remained under the critical photoperiod. The broilers were allowed access to food and water ad libitum. The diet was formulated to meet or exceed the nutrient recommendations for poultry, as outlined by the National Research Council (1994). At P14, blood samples were collected via cardiac puncture for ELISA, and then the birds were killed by decapitation. The left lobe of the liver was aseptically removed and frozen in liquid nitrogen and then stored at −80 °C for Western blot or quantitative real time PCR (QRT-PCR) analysis.

### 4.3. Primary Hepatocytes Isolation and Culture

The birds (*n* = 5) were anesthetized by the intravenous injection of a mixture of Nembutal (30–40 μg per gram of body weight) and heparin (1750 U/kg) at P14. The primary chick hepatocytes were isolated and cultured using a modification of the protocol published by Yamanaka [71] and Fraslin [72]. The abdominal cavity was opened under aseptic conditions to expose the liver. A catheter (Venocath, Abbott, Ireland) was inserted into the portal vein via the pancreaticoduodenal vein, and the right atriums were incised. The liver was first perfused with calcium-free HEPES solution (pH 7.4) supplemented with 5 mM of EDTA, and then the EDTA was washed off using a calcium-free HEPES solution, and the hepatocytes were obtained using HEPES solution containing 0.4 g/L of collagenase IV (Worthington Biochemical Corporation, Lakewood, CO, USA) and 5.4 mM of calcium chloride. The cell suspensions were filtered with a tissue sieve (200 meshes per 2.5 cm) and washed with William’s E medium (WMP02, Caisson, Smithfield, RI, USA) three times. The cells were re-suspended in William’s E medium with 10% fetal bovine serum (GIBCO, Carlsbad, CA, USA), and cell viability was assessed by trypan blue dye exclusion after isolation. Only preparations with a cell viability >95% were seeded into culture plates in an incubator at 37 °C. After 24 h of incubation, the supernatant was replaced by serum-free William’s E medium supplemented with 100 U/mL of penicillin, 0.1 mg/mL of streptomycin, 10^−9^ M of insulin (I0305000, Sigma, St. Louis, MO, USA), 10^−9^ M of dexamethasone (D4902, Sigma, St. Louis, MO, USA), 10^−7^ M of transferrin (T3309, Sigma, St. Louis, MO, USA), and 10^−5^ M of vitamin C (A7506, Sigma, St. Louis, MO, USA). The supernatant was renewed by serum-free William’s E medium every 24 h. Cells grown to 80–90% confluence were treated with 1 µM of prazosin (selective Mel1c antagonist, sc-204858, Santa Cruz, Dallas, TX, USA), 10 µM of PD98059 (MAP2K [MEK] 1/2 specific inhibitor, 1213, Bristol, Tocris Bioscience, MO, USA), 10 µM of Go9863 (a pan-PKC inhibitor, T6313, Topscience, TX, USA), or 10 µM of Ym254890 (an inhibitor of G_α_q/11 activation, 25700631, Wako, Osaka, Japan) for 30 min, followed by the addition of 250 pg/mL of MEL (63610, Sigma, St. Louis, MO, USA) or not for 24 h. All of the antagonist doses were determined by preliminary experiments.

### 4.4. ELISA

All of the blood samples were heparinized with 1000 UI/mL of sodium heparin (H4784, Sigma, St. Louis, MO, USA) in avian saline. After centrifugation at 1000× *g* for 20 min, the plasma was decanted and stored at −80 °C. Concentrations of MEL in plasma were measured by an enzyme-linked immunosorbent assay kit for anti-MEL (CEA908Ge, Uscn Life Science, Inc., Wuhan, China).

The cells were incubated with 1 μM of prazosin for 30 min followed by 250 pg/mL of MEL for 24 h, and 3-isobutyl-1-methylxanthin (IBMX 100 μM, T1713, Topscience, TX, USA) was added to the medium to prevent cAMP degradation before the cells were incubated for a further 30 min. The cells were collected and lysed with lysis buffer from the cAMP ELISA Kit (R&D Systems, Minneapolis, MN, USA). Further treatment of the samples was performed according to the manufacturer’s instructions.

The cells were incubated with 1 μM of prazosin or 10 μM of PD98059 for 30 min, followed by 250 pg/mL of MEL for 24 h. The supernatant was collected for IGF-I protein detection by the anti-chicken IGF-I ELISA kit (SEA050Ga, Uscn Life Science, Inc., Wuhan, China), according to the manufacturer’s protocols.

### 4.5. Quantitative Real-Time PCR (QRT-PCR)

The total RNA of the cell or tissue samples was extracted using the TRIzol reagent (CW0580A, CWBIO, Beijing, China), and immediately reverse transcribed using a reverse transcription kit (A5000, Promega, Madison, WI, USA). The expression levels of IGF-I and Mel1c were detected by QRT-PCR analysis, which was performed using the Roche LightCycler^®^96 real-time system (Software Version 1.1, Roche, Basel, Switzerland) with a SYBR Green Master Mix kit (Univ-bio, Shanghai, China). QRT-PCR analysis was performed under the following cyclic conditions for 40 cycles: 95 °C for 15 s (denaturation), 60 °C for 30 s (annealing), and 72 °C for 30 s (extension). The presence of a single PCR product was verified by melting curves in all of the amplifications. These data were analyzed by the comparative threshold cycle (CT) method and normalized against GAPDH controls. Primers were chosen from the Primer-BLAST website and are listed in Table 1. Relative mRNA expression was calculated by the 2^−∆∆*C*T^ method.

### 4.6. Western Blot Analysis

The hepatic tissues (left lobe) or hepatocytes were rapidly isolated and lysed in RIPA lysis buffer (CW2333S, CWBIO, Beijing, China) containing 1% protease inhibitor cocktail (CW2200S, CWBIO, Beijing, China) and 1% phosphatase inhibitor cocktail (CW2383S, CWBIO, Beijing, China). The lysates were centrifuged at 14,000× *g* for 15 min at 4 °C. The supernatants were collected, and the amount of protein was measured using a BCA protein assay Kit (CW0014, CWBIO, Beijing, China), before the protein concentration was standardized. Protein lysates were separated by electrophoresis in a 10% sodium dodecyl sulfate-polyacrylamide minigel (SDS-PAGE), and electrophoretically transferred to a polyvinylidene difluoride membrane (Millipore, Billerica, MA, USA). The membranes were first blocked with 5% fat-free milk for 1 h and then incubated overnight at 4 °C with the following specific antibodies: anti-phospho-ERK1/2 antibody or anti-ERK1/2 antibody (1:4000, M8159, M5670, Sigma, St. Louis, MO, USA), anti-phospho-PKC-pan (pThr497) antibody (1:500, SAB4504099, Sigma, St. Louis, MO, USA), anti-PKC antibody (1:200, sc-13149, Santa Cruz Biotechnology, Dallas, TX, USA), anti-G_α_q antibody (1:200, sc-365960, Santa Cruz Biotechnology, Dallas, TX, USA), and monoclonal mouse anti-β-actin antibody (CW0096, CWBIO, Beijing, China). The blot was incubated with horseradish peroxidase-conjugated (HRP) goat anti-rabbit or goat anti-mouse secondary antibodies (CW0103, CW0102, CWBIO, Beijing, China) at a dilution of 1:6000 for 1 h at 37 °C. The protein bands were detected using an enhanced chemiluminescence kit (CW0049, CWBIO, Beijing, China). The protein band intensities were quantified with Image J software version 1.41o (Sun Microsystems, Inc., Santa Clara, AL, USA). The relative phosphorylated protein level was quantified by the density ratio of phosphorylated protein to total protein. The relative G_α_q protein level was quantified by the density ratio to β-actin, and the relative protein level using the intact WL group in vivo or the control in vitro as 100%.

### 4.7. Data Analysis

The data were expressed as the mean ± standard error of the mean (SEM). The differences between the sham and surgical groups under the same light treatment were analyzed using independent sample T-tests. The differences among the various monochromatic lights within each operation group in vivo, and the differences among drug-treated groups in vitro were analyzed using one-way analysis of variance (one-way ANOVA) followed by Fisher’s least significant difference (LSD) post-hoc test using SPSS 16.0 software (SPSS Inc., Chicago, IL, USA). The level for determination of significance was *p* < 0.05.

## 5. Conclusions

Our results indicated that monochromatic lights influenced plasma MEL level and further regulated liver Mel1c expression of broiler chickens. The active Mel1c modulated hepatic IGF-I synthesis and secretion through coupling with G_α_q and activating PKC/ERK signaling pathway.

## Figures and Tables

**Table 1:** Primers used for QRT-PCR analysis.

Genes	Primer Sequences (5′-3′)	Product Size (bp)	Accession No.
IGF-I	F: TTCTTGAAGGTGAAGATGCACAC	217	NM_001004384.2
	R: TTCCCTTGTGGTGTAAGCGT		
Mel1c	F: AGA TAA GTG GGT TCC TGA TGG	237	NM_205361.1
	R: GCA AAG GTG CAA GAG TAA ATC		
GAPDH	F: ATC ACA GCC ACA CAG AAG ACG	124	NM_204305
	R:TGA CTT TCC CCA CAG CCT TA		

F = forward primer; R = reverse primer.

## Supplementary Materials

Supplementary materials can be found at https://www.mdpi.com/1422-0067/20/7/1682/s1.

## References

[1] Levenick C.K., Leighton A.T. (1988). Effects of photoperiod and filtered light on growth, reproduction, and mating behavior of turkeys. 1. growth performance of two lines of males and females. Poult. Sci..

[2] Zhang L., Zhang H.J., Qiao X., Yue H.Y., Wu S.G., Yao J.H., Qi G.H. (2012). Effect of monochromatic light stimuli during embryogenesis on muscular growth, chemical composition, and meat quality of breast muscle in male broilers. Poult. Sci..

[3] Rozenboim I., El Halawani M.E., Kashash Y., Piestun Y., Halevy O. (2013). The effect of monochromatic photostimulation on growth and development of broiler birds. Gen. Comp. Endocrinol..

[4] Halevy O., Biran I., Rozenboim I. (1998). Various light source treatments affect body and skeletal muscle growth by affecting skeletal muscle satellite cell proliferation in broilers. Comp. Biochem. Physiol. A Mol. Integr. Physiol..

[5] Wang T., Dong Y., Wang Z., Cao J., Chen Y. (2016). Secretion pathway of liver IGF-1 via JAK2/STAT3 in chick embryo under the monochromatic light. Growth Factors.

[6] Dishon L., Avitalcohen N., Malamud D., Heiblum R., Druyan S., Porter T.E., Gumulka M., Rozenboim I. (2017). In-ovo monochromatic green light photostimulation enhances embryonic somatotropic axis activity. Poult. Sci..

[7] Jones J.I., Clemmons D.R. (1995). Insulin-like growth factors and their binding proteins: Biological actions. Endocr. Rev..

[8] Ohlsson C., Mohan S., Sjögren K., Tivesten A., Isgaard J., Isaksson O., Jansson J.O., Svensson J. (2009). The role of liver-derived insulin-like growth factor-I. Endocr. Rev..

[9] Bai X., Wang Y., Wang Z., Cao J., Dong Y., Chen Y. (2016). In ovo exposure to monochromatic lights affect posthatch muscle growth and satellite cell proliferation of chicks: Role of IGF-1. Growth Factors.

[10] Liu W., Wang Z., Chen Y. (2010). Effects of monochromatic light on developmental changes in satellite cell population of pectoral muscle in broilers during early posthatch period. Anat. Rec. (Hoboken)..

[11] Haldar C., Ahmad R. (2010). Photoimmunomodulation and melatonin. J. Photochem. Photobiol. B.

[12] Park S.Y., Walker J.J., Johnson N.W., Zhao Z., Lightman S.L., Spiga F. (2013). Constant light disrupts the circadian rhythm of steroidogenic proteins in the rat adrenal gland. Mol. Cell Endocrinol..

[13] Claustrat B., Brun J., Chazot G. (2005). The basic physiology and pathophysiology of melatonin. Sleep Med. Rev..

[14] Pandi-Perumal S.R., Srinivasan V., Maestroni G.J., Cardinali D.P., Poeggeler B., Hardeland R. (2006). Melatonin: Nature’s most versatile biological signal. FEBS. J..

[15] Jin E., Jia L., Li J., Yang G., Wang Z., Cao J., Chen Y. (2011). Effect of monochromatic light on melatonin secretion and arylalkylamine N -Acetyltransferase mRNA expression in the retina and pineal gland of broilers. Anat. Rec..

[16] Li S., Cao J., Wang Z., Dong Y., Wang W., Chen Y. (2016). Melatonin mediates monochromatic light-induced Insulin-like growth factor 1 secretion of chick liver: Involvement of membrane receptors. Photochem Photobiol..

[17] Shiu S.Y., Ng N., Pang S.F. (1996). A molecular perspective of the genetic relationships of G-protein coupled melatonin receptor subtypes. J. Pineal Res..

[18] Jarzynka M.J., Passey D.K., Ignatius P.F., Melan M.A., Radio N.M., Jockers R., Rasenick M.M., Brydon L., Witt-Enderby P.A. (2010). Modulation of melatonin receptors and G-protein function by microtubules. J. Pineal Res..

[19] Shiu S.Y., Pang B., Tam C.W., Yao K.M. (2010). Signal transduction of receptor-mediated antiproliferative action of melatonin on human prostate epithelial cells involves dual activation of G_α_(s) and G_α_(q) proteins. J. Pineal Res..

[20] Zheng Y. (2004). G protein control of microtubule assembly. Annu. Rev. Cell Dev. Biol..

[21] Hepler J.R., Gilman A.G. (1992). G proteins. Trends. Biochem. Sci..

[22] Dhanasekaran N., Heasley L.E., Johnson G.L. (1995). G protein-coupled receptor systems involved in cell growth and oncogenesis. Endocr. Rev..

[23] Dhanasekaran N., Prasad M.V. (1998). G protein subunits and cell proliferation. Biol. Signals Recept..

[24] Rozengurt E. (1998). Signal transduction pathways in the mitogenic response to G protein-coupled neuropeptide receptor agonists. J. Cell. Physiol..

[25] Goldsmith Z.G., Dhanasekaran D.N. (2007). G protein regulation of MAPK networks. Oncogene.

[26] Simon M.I., Strathmann M.P., Gautam N. (1991). Diversity of G proteins in signal transduction. Science.

[27] Zhang L., Cao J., Wang Z., Dong Y., Chen Y. (2016). Melatonin modulates monochromatic light-induced GHRH expression in the hypothalamus and GH secretion in chicks. Acta. Histochem..

[28] Rozenboim I., Piestun Y., Mobarkey N., Barak M., Hoyzman A., Halevy O. (2004). Monochromatic light stimuli during embryogenesis enhance embryo development and posthatch growth. Poult. Sci..

[29] Allen R.E., Merkel R.A., Young R.B. (1979). Cellular aspects of muscle growth: Myogenic cell proliferation. J. Anim. Sci..

[30] Schaeffer H.J., Sirotkin A.V. (1997). Melatonin and serotonin regulate the release of insulin-like growth factor-I, oxytocin and progesterone by cultured human granulosa cells. Exp. Clin. Endocrinol. Diabetes..

[31] Schuster C., Gauer F., Guerrero H., Lakhdar-Ghazal N., Pevet P., Masson-Pevet M. (2000). Photic regulation of mt1 melatonin receptors in the Siberian hamster pars tuberalis and suprachiasmatic nuclei: Involvement of the circadian clock and intergeniculate leaflet. J. Neuroendocrinol..

[32] Schuster C., Gauer F., Malan A., Recio J., Pévet P., Masson-Pévet M. (2001). The circadian clock, light/dark cycle and melatonin are differentially involved in the expression of daily and photoperiodic variations in mt(1) melatonin receptors in the Siberian and Syrian hamsters. Neuroendocrinology.

[33] Masson-Pévet M., Gauer F., Schuster C., Guerrero H.Y. (2000). Photic regulation of mt(1) melatonin receptors and 2-iodomelatonin binding in the rat and Siberian hamster. Biol. Signals Recept..

[34] Kumar Yadav S., Haldar C., Kumar Singh S., Dash D. (2014). Melatonin regulates splenocytes proliferation via IP3-dependent intracellular Ca2+ release in seasonally breeding bird, Perdicula asiatica. J. Recept. Signal Transduct. Res..

[35] Bielawski K., Bielawska A., Sosnowska K., Miltyk W., Winnicka K., Pałka J. (2006). Novel amidine analogue of melphalan as a specific multifunctional inhibitor of growth and metabolism of human breast cancer cells. Biochem. Pharmacol..

[36] Mayo J.C., Hevia D., Quiros-Gonzalez I., Rodriguez-Garcia A., Gonzalez-Menendez P., Cepas V., Gonzalez-Pola I., Sainz R.M. (2017). IGFBP3 and MAPK/ERK signaling mediates melatonin-induced antitumor activity in prostate cancer. J. Pineal Res..

[37] Zhang J., Liu J., Ren L., Wei J., Zhang F., Li Y., Guo C., Duan J., Sun Z., Zhou X. (2018). Silica nanoparticles induce abnormal mitosis and apoptosis via PKC-δ mediated negative signaling pathway in GC-2 cells of mice. Chemosphere.

[38] Williams S.R., Son D.S., Terranova P.F. (2004). Protein kinase C δ is activated in mouse ovarian surface epithelial cancer cells by 2,3,7,8-tetrachlorodibenzo-p-dioxin (TCDD). Toxicology.

[39] Cardoso V.G., Gonçalves G.L., Costa-Pessoa J.M., Thieme K., Lins B.B., Casare F.A.M., de Ponte M.C., Camara N.O.S., Oliveira-Souza M. (2018). Angiotensin II-induced podocyte apoptosis is mediated by endoplasmic reticulum stress/PKC-δ/p38 MAPK pathway activation and trough increased Na + /H + exchanger isoform 1 activity. BMC Nephrol..

[40] Adornetto A., Pagliara V., Renzo G.D., Arcone R. (2013). Polychlorinated biphenyls impair dibutyryl cAMP-induced astrocytic differentiation in rat C6 glial cell line. Febs. Open Bio..

[41] Wu M., Jia J., Lei C., Ji L., Chen X., Sang H., Xiong L. (2015). Cannabinoid receptor CB1 is involved in nicotine-induced protection against Abeta1-42 neurotoxicity in HT22 cells. J. Mol. Neurosci..

[42] Renzi A., Glaser S., Demorrow S., Mancinelli R., Meng F., Franchitto A., Venter J., White M., Francis H., Han Y. (2011). Melatonin inhibits cholangiocyte hyperplasia in cholestatic rats by interaction with MT1 but not MT2 melatonin receptors. Am. J. Physiol. Gastrointest. Liver Physiol..

[43] Yung L.Y., Tsim S.T., Wong Y.H. (1995). Stimulation of cAMP accumulation by the cloned Xenopus melatonin receptor through G i and G z proteins. FEBS. Lett..

[44] Chan A.S., Lai F.P., Lo R.K., Voyno-Yasenetskaya T.A., Stanbridge E.J., Wong Y.H. (2002). Melatonin mt1 and MT2 receptors stimulate c-Jun N-terminal kinase via pertussis toxin-sensitive and -insensitive G proteins. Cell. Signal..

[45] Tam C.W., Shiu S.Y. (2011). Functional interplay between melatonin receptor-mediated antiproliferative signaling and androgen receptor signaling in human prostate epithelial cells: Potential implications for therapeutic strategies against prostate cancer. J. Pineal Res..

[46] Frey U.H., Lieb W., Erdmann J., Savidou D., Heusch G., Leineweber K., Jakob H., Hense H.W., Lowel H., Brockmeyer N.H. (2008). Characterization of the GNAQ promoter and association of increased Gq expression with cardiac hypertrophy in humans. Eur. Heart J..

[47] Kucukkaya B., Arslan D.O., Kan B. (2006). Role of G proteins and ERK activation in hemin-induced erythroid differentiation of K562 cells. Life Sci..

[48] Davis M.G., Kawai Y., Arinze I.J. (2000). Involvement of Gialpha2 in sodium butyrate-induced erythroblastic differentiation of K562 cells. Biochem J..

[49] Syrovatkina V., Alegre K.O., Dey R., Huang X.Y. (2016). Regulation, Signaling, and Physiological Functions of G-Proteins. J. Mol. Biol..

[50] Strange P.G. (2008). Signaling mechanisms of GPCR ligands. Curr. Opin. Drug Discov. Devel..

[51] Duc N.M., Kim H.R., Chung K.Y. (2015). Structural mechanism of G protein activation by G protein-coupled receptor. Eur. J. Pharmacol..

[52] Frank M., Thümer L., Lohse M.J., Bünemann M. (2005). G Protein activation without subunit dissociation depends on a G{alpha}(i)-specific region. J. Biol. Chem..

[53] Liu A.M., Ho M.K., Wong C.S., Chan J.H., Pau A.H., Wong Y.H. (2003). Galpha(16/z) chimeras efficiently link a wide range of G protein-coupled receptors to calcium mobilization. J. Biomol. Screen..

[54] Pingitore A., Chambers E.S., Hill T., Maldonado I.R., Liu B., Bewick G., Morrison D.J., Preston T., Wallis G.A., Tedford C. (2017). The diet-derived short chain fatty acid propionate improves beta-cell function in humans and stimulates insulin secretion from human islets in vitro. Diabetes Obes. Metab..

[55] Wang J., Carrillo J.J., Lin H.V. (2016). GPR142 agonists stimulate glucose-dependent insulin secretion via Gq-dependent signaling. PLoS ONE.

[56] Baba K., Benleulmi-Chaachoua A., Journé A.S., Kamal M., Guillaume J.L., Dussaud S., Gbahou F., Yettou K., Liu C., Contreras-Alcantara S. (2013). Heteromeric MT1/MT2 melatonin receptors modulate photoreceptor function. Sci. Signal..

[57] Ahmed R., Mahavadi S., Al-Shboul O., Bhattacharya S., Grider J.R., Murthy K.S. (2013). Characterization of signaling pathways coupled to melatonin receptors in gastrointestinal smooth muscle. Regul. Pept..

[58] Sotovega E., Meza I., Ramírezrodríguez G., Benitezking G. (2004). Melatonin stimulates calmodulin phosphorylation by protein kinase C. J. Pineal Res..

[59] Suzuki Y., Zhang H., Saito N., Kojima I., Urano T., Mogami H. (2006). Glucagon-like peptide 1 activates protein kinase C through Ca2+-dependent activation of phospholipase C in insulin-secreting cells. J. Biol. Chem..

[60] Jacobo S.M., Guerra M.L., Hockerman G.H. (2009). Cav1.2 and Cav1.3 are differentially coupled to glucagon-like peptide-1 potentiation of glucose-stimulated insulin secretion in the pancreatic beta-cell line INS-1. J. Pharmacol. Exp. Ther..

[61] Shigeto M., Ramracheya R., Tarasov A.I., Cha C.Y., Chibalina M.V., Hastoy B., Philippaert K., Reinbothe T., Rorsman N., Salehi A. (2015). GLP-1 stimulates insulin secretion by PKC-dependent TRPM4 and TRPM5 activation. J. Clin. Invest..

[62] Luttrell L.M. (2003). ‘Location, location, location’: Activation and targeting of MAP kinases by G protein-coupled receptors. J. Mol. Endocrinol..

[63] Osmond R.I., Sheehan A., Borowicz R., Barnett E., Harvey G., Turner C., Brown A., Crouch M.F., Dyer A.R. (2005). GPCR screening via ERK 1/2: A novel platform for screening G protein-coupled receptors. J. Biomol. Screen..

[64] Reindl K.M., Kittilson J.D., Bergan H.E., Sheridan M.A. (2011). Growth hormone-stimulated insulin-like growth factor-1 expression in rainbow trout (Oncorhynchus mykiss) hepatocytes is mediated by ERK, PI3K-AKT, and JAK-STAT. Am. J. Physiol. Regul. Integr. Comp. Physiol..

[65] Carbajo-Pescador S., García-Palomo A., Martín-Renedo J., Piva M., González-Gallego J., Mauriz J.L. (2011). Melatonin modulation of intracellular signaling pathways in hepatocarcinoma HepG2 cell line: Role of the MT1 receptor. J. Pineal Res..

[66] Wang T., Liu B., Guan Y., Gong M., Zhang W., Pan J., Liu Y., Liang R., Yuan Y., Ye L. (2018). Melatonin inhibits the proliferation of breast cancer cells induced by bisphenol A via targeting estrogen receptor-related pathways. Thorac. Cancer..

[67] Castro L.M., Gallant M., Niles L.P. (2005). Novel targets for valproic acid: Up-regulation of melatonin receptors and neurotrophic factors in C6 glioma cells. J. Neurochem..

[68] Tocharus C., Puriboriboon Y., Junmanee T., Tocharus J., Ekthuwapranee K., Govitrapong P. (2014). Melatonin enhances adult rat hippocampal progenitor cell proliferation via ERK signaling pathway through melatonin receptor. Neuroscience.

[69] Cao J., Liu W., Wang Z., Xie D., Jia L., Chen Y. (2008). Green and blue monochromatic lights promote growth and development of broilers via stimulating testosterone secretion and myofiber growth. J. Appl. Poult. Res..

[70] Karaganis S.P., Bartell P.A., Shende V.R., Moore A.F., Cassone V.M. (2009). Modulation of metabolic and clock gene mRNA rhythms by pineal and retinal circadian oscillators. Gen. Comp. Endocrinol..

[71] Yamanaka N., Kitani H., Mikami O., Nakajima Y., Miura K. (1997). Serum-free culture of adult chicken hepatocytes; morphological and biochemical characterisation. Res. Vet. Sci..

[72] Fraslin J.M., Touquette L., Douaire M., Menezo Y., Guillemot J.C., Mallard J. (1992). Isolation and long-term maintenance of differentiated adult chicken hepatocytes in primary culture. In Vitro Cell Dev. Biol..

