# Supplementary material for: Mel1c Mediated Monochromatic Light-Stimulated IGF-I Synthesis through the Intracellular Gαq/PKC/ERK Signaling Pathway
Source: Int J Mol Sci. 2019 Apr 4;20(7):1682. doi: 10.3390/ijms20071682 (PMC6480035; doi:10.3390/ijms20071682)
Supplement: Supplementary file 1 [file ijms-20-01682-s001.pdf]

Supplementary Materials: Mel1c protein expression was promoted by the treatment of different doses of melatonin for 24 hours

Shujie Ning, Zixu Wang, Jing Cao, Yulan Dong and Yaoxing Chen *

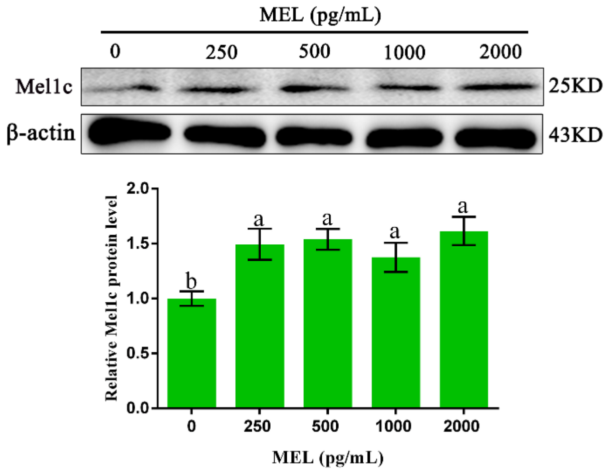

Figure S 1 Mel1c protein expression under stimulation of melatonin for 24 hours in hepatocytes of broiler. Hepatocytes were isolated from GL-treated intact broilers at P14 and incubated with 250 -2000 pg/mL MEL for 24 hours. The cells were then collected for Mel1c analysis by Western blot. Relative protein level was quantified by the density ratio of Mel1c protein to β-actin protein. Relative values were quantified using the control as 100%. Values with no common letters are significantly different ($p < 0.05$). MEL, melatonin.
